# Bringing light into darkness: peroral double-balloon enteroscopy-guided endoscopic injection sclerotherapy for a jejunal hemangioma

**DOI:** 10.1055/a-2437-8161

**Published:** 2024-11-22

**Authors:** Fu-qiang Liu, Wei Zhao, Yu-ting Huang, Xiangrong Zhou, Zhi-qiang Du

**Affiliations:** 1 546231 Department of Gastroenterology, The People’s Hospital of Jianyang City, Jianyang, China; 2 117972 Department of Gastroenterology, The First Affiliated Hospital of Chongqing Medical University, Chongqing, China

Small bowel bleeding accounts for 5–10% of all gastrointestinal bleeding cases, with small bowel hemangioma being one of the most common causes. It is characterized by an insidious onset and a high recurrence rate 1 . The standard treatment for small bowel hemangioma is surgical resection, which is both invasive and costly 2 . Recently, endoscopic injection sclerotherapy, typically used for esophageal varices, has been increasingly applied to treat gastrointestinal vascular lesions, including small bowel hemangioma 3 .

We report the case of a 52-year-old woman with no underlying systemic disease, who presented to our hospital with a 6-month history of recurrent melena and anemia. Laboratory tests revealed iron deficiency anemia, with a hemoglobin level at 6.9 g/dL. Contrast-enhanced abdominal computed tomography, esophagogastroduodenoscopy, and colonoscopy did not identify a bleeding source. Subsequently, peroral double-balloon enteroscopy revealed a 2.0-cm irregular, elevated lesion in the middle of the jejunum. The lesion was soft and non-pulsatile, consistent with the appearance of a hemangioma ( Fig. 1 **a** , Video 1 ). Bleeding points were identified in the central depression of the lesion, indicating the bleeding source ( Fig. 1 **b** ). Methylene blue dye was applied to delineate the margins of the lesion ( Fig. 1 **c** ). After confirming positive blood reflux, we performed endoscopic injection sclerotherapy using an 8-ml polidocanol-methylene blue mixture ( Fig. 1 **d** ). After the injection, the hemangioma was notably reduced in size ( Fig. 1 **e** ). For future radiological or surgical reference, a metal clip was anchored to the proximal part of the jejunum, 5 cm away from the lesion ( Fig. 1 **f** ). The patient remained free of recurrence during the 1-year follow-up.

Endoscopic injection sclerotherapy for a hemangioma located in the middle part of the jejunum using peroral double-balloon enteroscopy in a 52-year-old woman with recurrent melena and anemia.Video 1

**Fig. 1:**
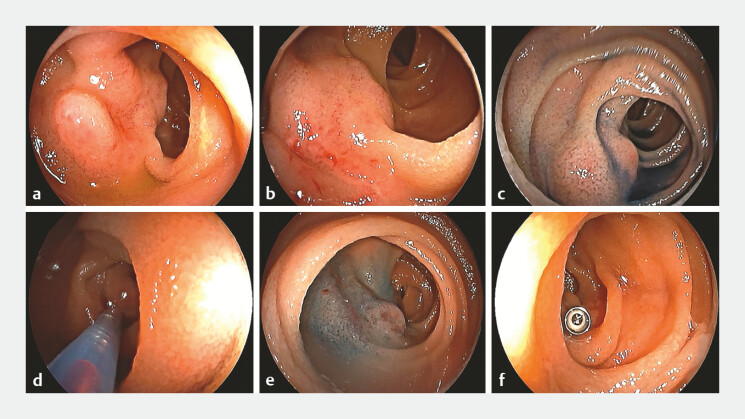
Peroral double-balloon enteroscopy-guided endoscopic injection sclerotherapy for a hemangioma located in the middle part of the jejunum. **a** Double-balloon enteroscopy showing an irregular elevated lesion in the middle part of the jejunum. **b** Bleeding spots located in the central depressed part of the lesion, indicating the bleeding source. **c** Methylene blue was applied to the lesion to delineate its margins. **d** Positive blood reflux was seen within the puncture needle. **e** After the injection, the hemangioma was reduced in size. **f** For future radiological or surgical reference, a metal clip was anchored to the jejunal wall in the proximal part of the jejunum, 5 cm away from the lesion.

Endoscopic intervention for small bowel hemangioma poses significant challenges due to anatomical and technical limitations. Although various endoscopic treatments, such as double-balloon enteroscopy-guided argon plasma coagulation, polypectomy, and endoscopic mucosal resection, have been proposed for small bowel hemangioma, a definitive consensus on the optimal approach is lacking 2 4 . Based on our experience, double-balloon enteroscopy-guided endoscopic injection sclerotherapy is a simple, effective, convenient, and minimally invasive procedure that requires only a standard injection needle. It should be considered a preferred treatment option for small bowel hemangioma, particularly in patients with recurrent episodes or those who are unsuitable for surgery.

Endoscopy_UCTN_Code_TTT_1AP_2AD

## Notes

Endoscopy E-Videos https://eref.thieme.de/e-videos
*E-Videos* is an open access online section of the journal *Endoscopy* , reporting on interesting cases and new techniques in gastroenterological endoscopy. All papers include a high-quality video and are published with a Creative Commons CC-BY license. Endoscopy E-Videos qualify for HINARI discounts and waivers and eligibility is automatically checked during the submission process. We grant 100% waivers to articles whose corresponding authors are based in Group A countries and 50% waivers to those who are based in Group B countries as classified by Research4Life (see: https://www.research4life.org/access/eligibility/ ). This section has its own submission website at https://mc.manuscriptcentral.com/e-videos .
